# Editorial: Reviews on the effect of COVID-19 on kidney diseases diagnosis, management, and outcomes

**DOI:** 10.3389/fmed.2023.1148402

**Published:** 2023-03-10

**Authors:** Kam Wa Chan, Nianqiao Gong, Bassam G. Abu Jawdeh

**Affiliations:** ^1^Department of Medicine, The University of Hong Kong, Pokfulam, Hong Kong SAR, China; ^2^Institute of Organ Transplantation, Tongji Hospital, Tongji Medical College, Huazhong University of Science and Technology, Wuhan, China; ^3^Division of Nephrology, Mayo Clinic, Phoenix, AZ, United States

**Keywords:** COVID, acute kidney injury, chronic kidney disease, transplant, dialysis

COVID-19 causes acute kidney injury (AKI) with varied incidence globally (1, 2). The basal prevalence of chronic kidney disease (CKD) associates with AKI incidence at the population level, which further correlates with prolonged hospitalization and mortality (2). Only a handful of settings had surveillance of kidney health before the outbreak, complicating the early detection and management of AKI. Also, the translation of the pathophysiological understanding of AKI to effective management regimens is limited. In his book, *The Tipping Point*, Malcolm Gladwell discusses “the stickiness factor” as a major driver of epidemics. There is no better example than immunosuppression as the “stickiness factor” contributing to worse outcomes in advanced kidney disease patients and immunosuppressed transplant recipients. Transplant-related immunosuppression is associated with lower seropositivity after vaccination, which requires dedicated and personalized management (3). In this issue, the papers addressed and summarized these key aspects pertinent to kidney and transplant patients.

The systematic review by Mallhi et al. once again highlighted the heterogeneity and clinical implication of AKI in different populations. Legouis et al. applied machine learning methods to cluster patients into three subphenotypes based on the use of medications at admission, baseline kidney function and severity of the condition. The three clusters were correlated with different severity of clinical presentation, metabolic profile, and risk of hospital death, which could represent different underlying pathophysiology of AKI. Pacheco et al. summarized the phenotypes of kidney injury in setting of COVID-19. Although hemodynamic-mediated AKI in systemic infection remains the most common, multiple glomerulopathies have been implicated. Collapsing glomerulopathy is the most prevalent, however a wide range of pathologies have been reported throughout the pandemic associating SARS-CoV-2 infection with almost every glomerular disease that exists.

A Thai nationwide peritoneal dialysis cohort was analyzed by Chuengsaman et al.. Functional capacity, and the need of respiratory support remain as major risk factors that compound the kidney disease status, and augment mortality. Despite a theoretical decreased exposure to the SARS-CoV-2 virus, peritoneal dialysis patients experienced a high mortality rate comparable to hemodialysis and transplant patients. This emphasizes the vulnerability of the kidney failure patients to infections.

The already complex kidney transplant process was further complicated by the pandemic. Zhang et al. eloquently described the evolution of transplant operations throughout the COVID-19 pandemic, starting from suspending transplant programs all-together, to currently, utilizing kidney allografts from deceased donors who died due to active SARS-CoV-2 infection. This highlights the improved comfort level dealing with the pandemic as we got to understand more about the virus, especially as vaccinations became more widespread and therapeutic options became available. The authors also discuss some of the implementation changes from pre-operative evaluations, perioperative care to post-transplantation maintenance, that are now here to stay beyond the pandemic. This applies mostly to the utilization of virtual platforms which have proven to improve efficiency and reduce cost for both patients and healthcare organizations.

Vaccination helps boost polyfunctional CD4+ T cell response to protect our vulnerable kidney patients against severe COVID-19. Nevertheless, Kodali et al. and Wiedemann et al. both highlighted the diminished response to vaccines in kidney transplant recipients on immunosuppression particularly belatacept-based regimens, where both the humoral and cellular pathways are significantly dampened. There is an unmet need for systematically investigating passive immunization and heterologous vaccination strategies in this subgroup with close monitoring. The “holy grail” in the acute setting appears to be temporarily reducing the intensity of immunosuppression cautiously and in an individualized fashion. Interestingly, COVID-19 infection or vaccination does not necessarily associate with increased rejection risk (4). Although vaccines altered the course of the pandemic favorably, they have been associated with multiple histopathologic phenotypes, including IgA-nephropathy, minimal change disease, pauci-immune glomerulonephritis in addition to others. Despite the plausible causality given that vaccines stimulate the immune system, existing data remains limited to raise concern against vaccination, the cornerstone for battling the COVID-19 pandemic.

In this series, we better characterized AKI from the perspective of epidemiology and pathophysiology through meta-analysis and machine learning. COVID-19 led to the optimization of telemedicine in the management of transplantation. Vaccine introduction has been game-changing from the public health perspective. The spotlight is now on the vulnerable groups who could not fully benefit from the mass intervention. In transplant patients, the strategy of maximizing immunity gain by vaccination and adjusting basal immunosuppression would require personalized consideration. Perhaps the most important lesson learned from the COVID-19 pandemic, is the need to develop an adequate infrastructure, dialysis, and immunosuppression management protocols for prompt and effective handling of any future pandemics.

## Author contributions

All authors listed have made a substantial, direct, and intellectual contribution to the work and approved it for publication.
